# Tetravalent Influenza Vaccine Is Not Associated With Neuroaxonal Damage in Multiple Sclerosis Patients

**DOI:** 10.3389/fimmu.2021.718895

**Published:** 2021-08-26

**Authors:** Tobias Moser, Michael Seiberl, Julia Feige, Lara Bieler, Richard F. Radlberger, Ciara O’Sullivan, Georg Pilz, Andrea Harrer, Kerstin Schwenker, Elisabeth Haschke-Becher, Lukas Machegger, Jochen Grimm, Monika Redlberger-Fritz, Arabella Buchmann, Michael Khalil, Erich Kvas, Eugen Trinka, Peter Wipfler

**Affiliations:** ^1^Department of Neurology, Christian Doppler University Hospital, Paracelsus Medical University and Center for Cognitive Neuroscience, Salzburg, Austria; ^2^Department of Laboratory Medicine, Paracelsus Medical Center, Salzburg, Austria; ^3^Department of Neuroradiology, Christian Doppler University Hospital, Paracelsus Medical University, Salzburg, Austria; ^4^Center for Virology, Medical University of Vienna, Vienna, Austria; ^5^Department of Neurology, Medical University of Graz, Graz, Austria; ^6^Hermesoft, Statistics, Graz, Austria; ^7^Neuroscience Institute, Christian Doppler University Hospital, Paracelsus Medical University and Center for Cognitive Neuroscience, Salzburg, Austria

**Keywords:** vaccination, immunization, NfL, titers, influenza, COVID-19, antibody response

## Abstract

**Background:**

Efficacy of vaccines and disease activity linked to immunization are major concerns among people with multiple sclerosis (pwMS).

**Objective:**

To assess antibody responses to seasonal influenza antigens and vaccine-associated neuroaxonal damage utilizing serum neurofilament light chain (sNfL) in pwMS receiving dimethyl fumarate (DMF).

**Methods:**

In this prospective study, the 2020/2021 seasonal tetravalent influenza vaccine was administered to 20 pwMS treated with DMF and 15 healthy controls (HCs). The primary endpoints were responder rate of strain-specific antibody production (seroconversion or significant (4-fold) increase in influenza-antibody titers for ≥2/4 strains) at 30 days post-vaccination and changes in sNfL levels.

**Results:**

All patients treated with DMF fulfilled the responder criteria for immunization compared with 53% of the controls. However, higher proportions of HCs already had influenza-antibody titers ≥1:40 at baseline (53% *vs.* 41%, p = 0.174). sNfL levels were comparable among both groups at baseline and did not increase 34 days after vaccination. In addition, no clinical or radiological disease reactivation was found.

**Conclusion:**

DMF-treated patients mount an adequate humoral immune response to influenza vaccines. Within the limits of the small cohort investigated, our data suggest that influenza immunization is not associated with clinical or subclinical disease reactivation.

## Introduction

Vaccines are a major achievement of science, and many of them protect from life-threatening infectious disease or have eradicated global health threats such as smallpox (1). However, false studies or misguided reports, including lay advice on social media, impact public attitude and contribute to the growing vaccine hesitancy (2, 3). Anti-vaccine sentiments constitute a major obstacle in times when immunization coverage is crucial to combatting a global pandemic. People suffering from chronic immune-mediated disorders including multiple sclerosis (MS) are confronted with particular vaccine-related concerns. First, people with MS (pwMS) are more susceptible to infections (4) mainly due to suppressive properties of disease-modifying therapies (DMTs) on normal immune functions (5). Infections, on the other hand, frequently trigger neurological deteriorations, which have been reported to be more severe than spontaneous relapses (6, 7). Immunizations hence not only are essential to prevent infections but may even be considered neuroprotective in MS. In fact, the seasonal influenza vaccine is highly recommended for MS patients (8). Another concern is that the immunomodulating/immunosuppressive effects of MS drugs may reduce vaccine efficacy. Trials in this regard have primarily assessed humoral responses to seasonal influenza vaccinations. Findings indicate that, apart from beta-interferons, many DMTs diminish immune responses to vaccinations (9). For dimethyl fumarate (DMF), one of the most frequently used MS therapeutics (10), humoral response against bacterial antigens was shown to be preserved (11), but data on vaccine efficacy to viral pathogens is completely lacking.

Irrespective of infectious concerns, patients also fear neurological sequelae following immunization, again fostering vaccine reluctance in MS. Several reports have found no link between seasonal flu vaccination and MS exacerbation assessed by the current standard of care with magnetic resonance imaging (MRI) scans and/or clinical examination (12–16), while two small studies could not refute an association (17, 18).

More recently, serum neurofilament light chain (sNfL) has been proposed as a biomarker for neuronal injury (19). NfL represents a major constituent of the axonal cytoskeleton in neurons, and it is released into the cerebrospinal fluid and the peripheral circulation upon neuro-axonal injury. In fact, a growing body of evidence supports its role as surrogate for disease activity and potentially for subclinical neuro-axonal damage (19–22). sNfL therefore appears to be a sensitive marker to unveil contrasting results from the literature regarding the impact of vaccines on disease activity.

The aim of this study was to elucidate whether vaccine-induced immunological consequences are linked to increases in sNfL in pwMS. Moreover, we investigated whether the immunomodulating properties of DMF blunt the efficacy of viral vaccines.

## Materials and Methods

We conducted a prospective study to assess efficacy and safety of seasonal flu immunization in MS patients treated with DMF. We recruited patients aged 18 to 65 with relapsing MS according to the McDonald criteria 2017 (23) from an outpatient MS clinic in a large university center and 15 healthy controls (HCs). Eligible patients were required to receive DMF in the approved dose (240 mg twice daily) for at least 3 months and have not been treated with steroids within 4 weeks from vaccination. Patients with prior immunosuppressive drugs or concomitant, clinically significant systemic diseases at baseline (BL) were excluded.

### Assessments

Serum samples for each individual were drawn before and 4 weeks after vaccination and stored at −80°C. Participants were immunized with injectable seasonal influenza vaccines 2020/2021 (VaxigripTetra^®^, Sanofi Pasteur Europe; or Influvac Tetra^®^, Mylan Healthcare GmbH) comprising antigens of A(H1N1)pdm09 A/Guangdong-Maonan/SWL1536/2019, A(H3N2) A/Hong Kong/2671/2019, B/Victoria lineage B/Washington/02/2019 (B/Vic), and B/Yamagata lineage B/Phuket/3073/2013 (B/Yam) strains (**Table 1**). Both vaccines contained 15 µg of each strain. Amounts of strain-specific antibodies were quantitatively obtained by hemagglutination inhibition assay (HAI). HAIs were performed blinded and in duplicates. Disease activity following influenza vaccination was gauged by clinical, radiological, and laboratory parameters. Clinical and sNfL evaluations were determined just prior to vaccination (BL) and 30 days thereafter. sNfL concentrations were assessed by a commercially available single-molecule array (SIMOA) assay NF-light^®^ kit on the SR-X Analyzer (Quanterix, Lexington, MA). Cerebral MRI (cMRI) analyses were performed on 3-tesla MRIs 4 weeks post-vaccination and compared with the most recent pre-immunization image carried out on the same scanner. All cMRIs included T1-weighted images before and after administration of contrast agent [gadolinium (Gd)] and T2/fluid-attenuated inversion recovery (FLAIR) sequences. Images were analyzed by two independent neuroradiologists (JG and LM).

**Table 1:** Demographics and clinical data.

	DMF 2 × 240 mg (n = 20)	HC (n = 15)
**Age, mean (** ± **SD)**	37.6 (8.5)	36.3 (10)
**Sex, no. (%)**		
Female	12 (60)	12 (80)
Male	8 (40)	3 (20)
**EDSS, median (IQR)**		
Baseline	1.0 (0–1.5)	–
Follow-up	1.0 (0–1.5)	–
**Months since MS diagnosis, mean (** ± **SD)**	39.3 (59.1)	–
**Months since DMF start, mean (** ± **SD)**	19.2 (12.1)	–
**Annualized relapse rate 12 months before screening**	0.25	–
**DMT before DMF**		
None	18	–
IFN	2	–
**Vaccine, no. (%)**		
VaxigripTetra	2 (10)	15 (100)
Influvac Tetra	18 (90)	–
**Mean time from vaccination to follow-up in days ( ± SD)**	29.1 (2.9)	40.7 (10.8)
**Lymphocyte count, no.**		
>0.91 × 10^3^/L	15	–
<0.91 × 10^3^/L	2	–
<0.80 × 10^3^/L	3	–
<0.50 × 10^3^/L	0	–

DMF, dimethyl fumarate; HC, healthy control; no., number; IFN, interferon beta; DMT, disease-modifying therapy; SD, standard deviation; IQR, interquartile range; EDSS, Expanded Disability Status Scale.

### Study Endpoints

The primary influenza-vaccine efficacy outcome was responder rate at 30 days from immunization in accordance with European Guidelines (24). The responder rate was determined as the proportion of individuals to fulfill either the criteria for seroconversion or a significant antibody increase for at least two of the four influenza strains. Participants who had BL titers of ≤1:10 and after immunization reached the cut-off for seroprotection were defined as seroconverters. An antibody increase by 4-fold was considered significant based on regulatory guidelines for vaccination trials. Seroprotection was defined as a HAI of ≥1:40 according to literature recommendations (25). The primary safety outcome was determined by sNfL. Also, relapses and Expanded Disability Status Scale (EDSS) changes during the study period were assessed by MS specialists. Post-vaccine cMRI scans were primarily evaluated for Gd enhancement but also for new/enlarging lesions compared with pre-vaccine MRI. In addition, we assessed routine laboratory parameters within the MS cohort including inflammatory proteins (IL-6 and C-reactive protein (CRP)) and main immune cell subsets.

### Statistical Analysis

This project was an exploratory study, and therefore, all analyses have to be seen on a descriptive level. After testing for normality using the Kolmogorov–Smirnov test, results were presented as median [interquartile range (IQR)] and/or mean ± standard deviation (SD), as appropriate. Qualitative variables are shown as absolute counts and/or percentages.

Quantification of sNfL levels pre- and post-vaccination as well as the comparison between MS patients and HCs was investigated using the Wilcoxon signed-rank test for changes over time and the Mann–Whitney U or Fisher test (as appropriate) to compare groups at each time point. All other quantitative variables were analyzed according to the primary objective.

Statistical testing and 95% confidence intervals were used to detect possible signals and not to confirm planned hypotheses. Significance levels were set at nominal p-values of p ≤ 0.05, and no corrections for multiple testing were performed. Statistical analyses were done using IBM SPSS Statistics Version 24 (IBM Corp., Armonk, NY, USA). Graphs were designed by GraphPad PRISM8 (GraphPad Software, San Diego, CA, USA).

### Ethics

The study was approved by the local ethics committee (415-E/1612/11-2018) and conducted according to the Good Clinical Practice and the ethical principles of the Declaration of Helsinki. All participants provided written informed consent.

## Results

### Baseline Demographics and Pre-Vaccine Seroprotection Rates

We enrolled 20 pwMS treated with DMF and 15 age-matched controls. The mean age of the MS cohort was 37.6 years ( ± 9), with a median EDSS at BL of 1.0 (IQR 0–1.5). Patients had received DMF on average for 19.2 months ( ± 12.1). The demographics and vaccine distribution of the two cohorts and the EDSS score of the MS cohort are displayed in **Table 1**. The time from vaccination to the follow-up visit was longer for HCs (means 40.7 *vs.* 29.1 days, p < 0.05).

**Figure 1:**
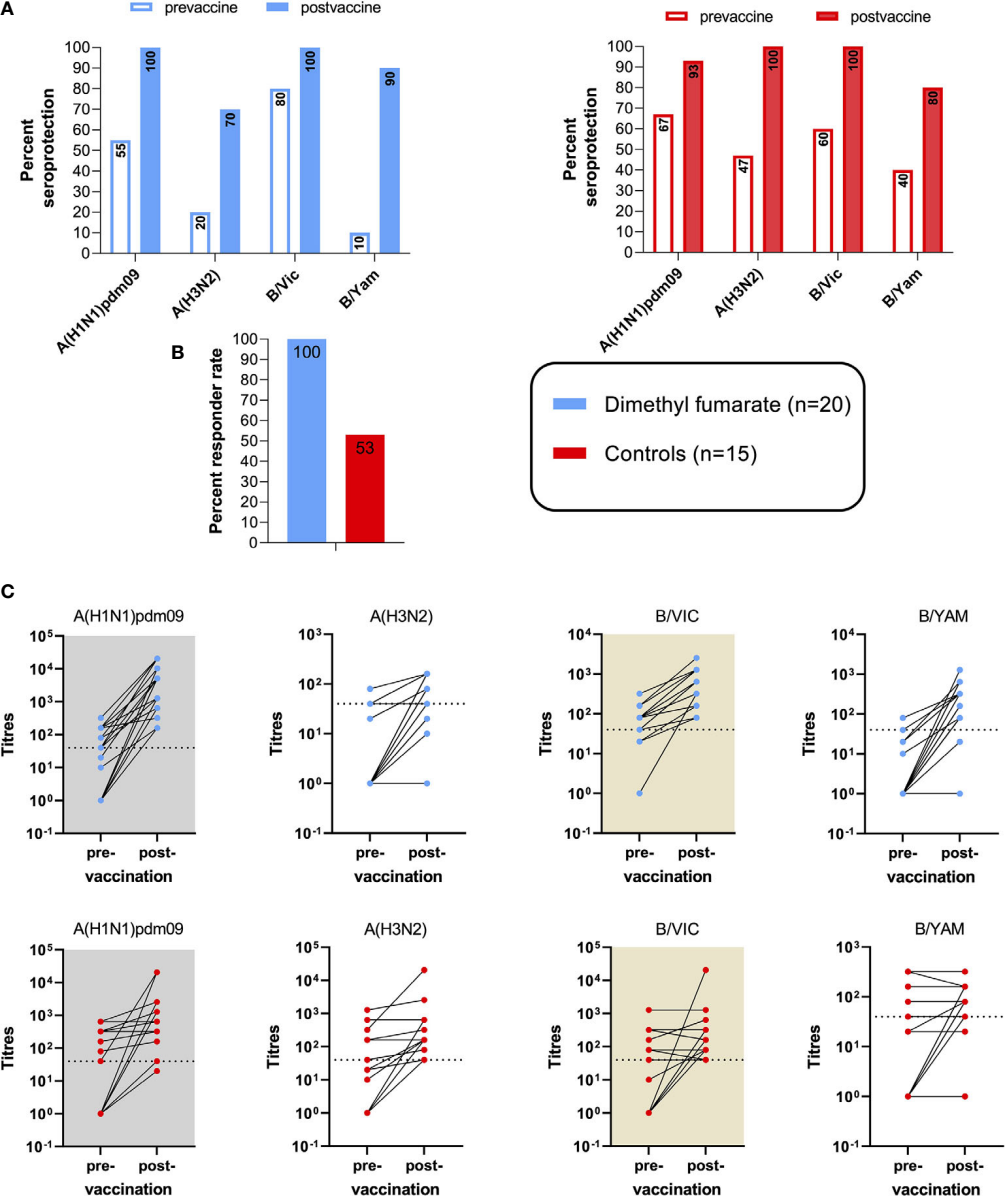
Vaccine efficacy to influenza immunization in multiple sclerosis (MS) patients on dimethyl fumarate (DMF) and healthy controls. **(A)** Pre- and post-vaccine seroprotection rates, defined by a strain-specific anti-influenza titer of ≥1:40. **(B)** Vaccine responder rates among the two cohorts. Vaccine response was defined by seroconversion and/or significant (≥4-fold) specific titer increases in ≥2/4 influenza strains. **(C)** Increases in strain-specific antibody titers among the two cohorts at 34.1 days ( ± 9.4) post-vaccination compared with baseline. Dotted lines indicate the cut-off titer for seroprotection.

At BL, seroprotection (antibody titers ≥1:40) was more frequent among HCs in three of the four influenza strains (**Figure 1A**). Across all strains, the proportions of patients *vs.* controls who met the criteria for seroprotection was 41% *vs.* 53% (p = 0.174), respectively. The cut-off was evident in patients *vs.* controls in 55% (11/20) and 66% (10/15) for A(H1N1)pdm09, in 20% (4/20) and 47% (7/15) for A(H3N2), in 80% (16/20) and 60% (9/15) for B/Vic, and in 10% (2/20) and 40% (6/15) for B/Yam.

At BL, average A(H3N2) titers were higher in the control group (p = 0.007), while no significant differences were found among the other strains.

### Vaccine Efficacy

The primary efficacy endpoint was responder rate at 30 days from vaccination. This outcome was reached in 100% of DMF-treated patients, compared with 53% of controls (**Figure 1B**).

A seroconversion and/or 4-fold increase in antibody titers for DMF-treated MS patients *vs.* controls was achieved in 95% *vs.* 47% for A(H1N1)pdm09, in 60% *vs.* 40% for A(H3N2), in 85% *vs.* 53% for B/Vic, and in 90% *vs.* 40% for B/Yam. The increases in strain-specific antibody titers are shown in **Figure 1C**. Over all strains, a significant (4-fold) increase in antibody titers for DMF-treated MS patients and controls was found in 49% and 15%, respectively; and the criteria for seroconversion were met by 36% and 30%, respectively.

Thirty days post-vaccination, seroprotection was evident in 100% (20/20) and 93% (14/15) for A(H1N1)pdm09, in 70% (14/20) and 100% (15/15) for A(H3N2), in 100% (20/20) and 100% (15/15) for B/Vic, and in 90% (18/20) and 80% (12/15) for B/Yam in DMF patients and controls, respectively (**Figure 1A**). Across all four strains, seroprotection was reached by 90% of MS patients and by 93% of the controls at follow-up.

At follow-up, the increase of average antibody levels was statistically significant for MS and HCs against A(H1N1)pdm09 (p < 0.001 and p = 0.003, respectively), against A(H3N2) (p < 0.001 and p = 0.001, respectively), and against B/Vic (p < 0.001 and p = 0.050, respectively). For the B/Yam strain, a statistically significant increase was found only among the MS cohort (p < 0.001). Regarding inter-group differences of humoral vaccine responses, average titer increases against A(H1N1)pdm09, B/Vic, and B/Yam were more pronounced among the MS group (p = 0.014, p = 0.003, and p < 0.001, respectively).

### Disease Activity

No relapses or neurological deteriorations were reported within the observational period of 4 weeks after vaccination. Also, post-vaccination cMRI scans did not show any Gd-enhancing lesions. Compared with the most recent pre-immunization MRI scan (28.4 ± 18.8 weeks apart from BL), no new or enlarging lesions were found in 16 patients (80%). Four patients (20%) showed one or two new FLAIR hyperintense lesions as compared with pre-vaccine MRI (41 ± 12.6 weeks apart).

**Figure 2:**
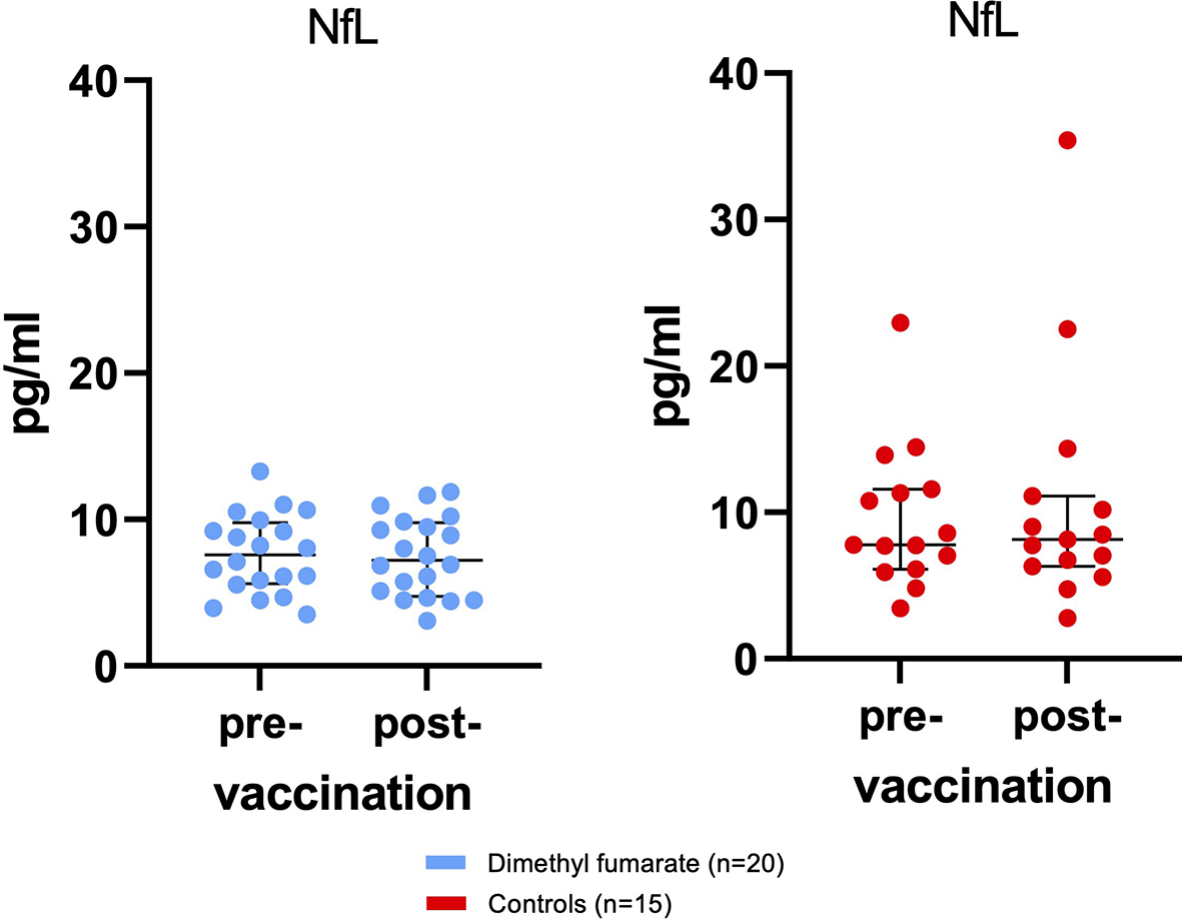
No subclinical disease activity as measured by serum neurofilament light chain (sNfL) associated with influenza vaccination was found. Displayed are sNfL values for patients and controls at baseline and 34.1 days ( ± 9.4) after immunization with influenza vaccine. Bars indicate median and interquartile range (IQR).

The primary safety variable was vaccine-associated increases in sNfL (**Figure 2**). Mean pre-vaccine NfL levels from MS patients were 7.64 pg/ml ( ± 2.67 pg/ml) and did not increase 4 weeks after immunization (7.5 ± 2.7 pg/ml). There were no significant differences in sNfL levels between pwMS and HCs (BL: 9.6 ± 4.9 pg/ml; follow-up: 10.7 ± 8.3 pg/ml) at either measuring point.

During the investigational period, two controls and one MS patient suffered from a COVID-19 infection (two SARS-CoV-2 PCR confirmed cases (one in each cohort) and one probable case without laboratory test among the HCs). NfL levels of the two controls increased above average by >10 pg/ml (from 11.3 to 22.5 pg/ml and from 22.9 to 35.4 pg/ml), while sNfL from the MS patient on DMF remained stable (from 6.1 to 5.1 pg/ml).

### Routine Laboratory Findings

**Figure 3:**
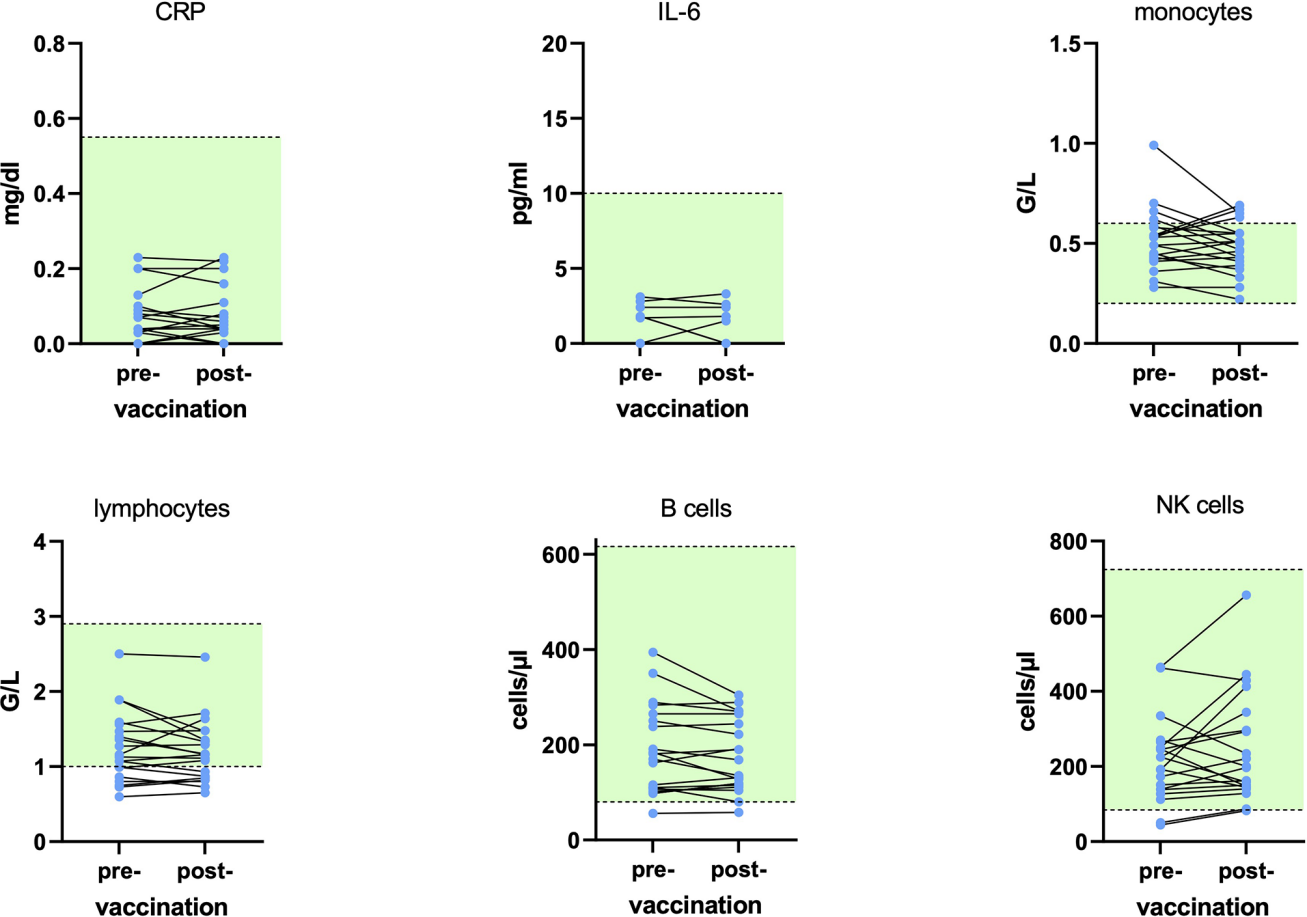
Course of inflammatory proteins and main immune cells at baseline and 29.1 ( ± 2.9) days after influenza vaccination for 20 patients on dimethyl fumarate (DMF). No statistically significant alterations were found. Green areas display the reference range. CRP, C-reactive protein; IL-6, interleukin 6; NK, natural killer.

Additional laboratory parameters were assessed at BL and 4 weeks after immunization for the pwMS. No patient exhibited either Grade 3 or 4 lymphopenia, while Grade 2 lymphopenia was found in four patients (20%). CD19+ B cells were below the limit of normal in 5% (1/20). The courses of the main immune subsets and inflammatory parameters (interleukin 6 and CRP) are displayed in **Figure 3**.

### Tolerability

There were no safety concerns for either cohort in this study. The seasonal influenza vaccine was well tolerated by all participants.

## Discussion

This pilot study assessing efficacy and safety of influenza vaccination in 20 MS patients treated with DMF reports on two key findings. First, despite its multiple immunomodulatory effects (26), DMF preserves humoral immune responses to specific viral antigens. In fact, we observed a 100% responder rate to tetravalent 2020/2021 influenza immunizations in DMF-treated patients, which was even higher than that of controls. This is likely influenced by the responder rate definition, since controls have had higher pre-vaccine seroprotection rates (53% *vs.* 41%). Across all strains, seroprotection increased to 93% and 90% for controls versus pwMS after immunization. Moreover, the period from vaccination to titer assessment was longer for controls. Less likely, the usage of two vaccines from different companies (both containing the same strains and doses) impacted on the outcome. In line with our results, vaccine efficacy to bacterial antigens was also shown to be adequate in DMF-treated pwMS (11). Together, the mode of action of DMF appears not to interfere with specific antibody production, assuming that immune responses to other vaccines, including COVID-19 mRNA and adenovirus vectors, to deliver immunization would be preserved. Unimpaired immune functions to control pathogens are also supported by the fact that DMF treatment, despite induction of lymphopenia, is not linked to an increased risk of infections (27, 28). In contrast to adequate humoral immune functions under DMF, diminished vaccine responses have been reported for several DMTs including fingolimod, glatiramer acetate, natalizumab, and CD20-depleting agents (29–31).

In accordance with prior studies, we confirm that seasonal influenza vaccination is not linked to clinical or radiological deteriorations in MS (12–16). In addition, our study is, to the best of our knowledge, the first to show that immunization is not associated with an increase in sNfL in DMF-treated pwMS or in HCs. This is crucial, as NfL represents a specific biomarker for neuro-axonal cell damage, able to detect even discreet, subclinical neuroinflammation leading to neuro-axonal injury. Moreover, NfL increase not only is restricted to axonal damage of the brain but also reflects pathology within the spinal cord (19). In contrast to the limited ability of MRI scans to detect ongoing (sub-)clinical inflammation of the gray matter and neuro-axonal degeneration, NfL can serve as a holistic biomarker for disease activity (19).

Our data support the rationale that vaccine-induced processes are restricted to the peripheral immune system without comprising the functionality of the blood–brain barrier and therefore not precipitating inflammation in the CNS of MS patients. This is crucial, as immune reactions associated with systemic infections appear to impact the integrity of the CNS, eventually resulting in MS exacerbations (6, 7). In fact, recent studies revealed elevated NfL levels during COVID-19, irrespective of the clinical course (32, 33). Moreover, increased NfL concentrations at the time of admission of COVID-19 patients were linked to a higher mortality risk (34). This is of interest as SARS-CoV-2 primarily affects the respiratory system, and a direct impact on neurons has not ultimately been clarified (35–37). In line with these reports, we found relevant increases in sNfL among two controls infected by SARS-CoV-2. Intriguingly, NfL from the DMF patient who also suffered from COVID-19 during the study period remained stable. To date, sNfL levels during systemic infections other than COVID-19 have not been extensively studied. However, the reports on NfL increases during COVID-19 together with our findings on safety and efficacy of vaccines argue that, particularly in light of the current pandemic, preventing infections by immunization should therefore be strongly considered in vulnerable populations like MS patients.

The small number of patients enrolled mainly limits our study, and the results must be interpreted in this context. Another limitation is that no specific BL MRI and no spinal cord images were available, with the most recent cMRI prior to vaccination being used for comparison. Also, the observational period of 4 weeks post-immunization appears short for clinical evaluations, but as sNfL assessment was the primary outcome parameter, the interval was considered appropriate. However, we cannot ultimately rule out disease reactivation after the follow-up period. Considering data from the literature, we strongly believe, that a) disease activity associated with vaccination, if any, would occur within 4 weeks and b) sNfL would increase within this period similar to early increases found in small vessel disease (38) and traumatic brain injuries (39–41). sNfL is currently considered the most appropriate serum biomarker for neuroaxonal damage, yet stable values cannot exclude neuroaxonal pathology with absolute certainty. Even though we found no clinical and serological evidence for neurological damages associated with immunization, safe administration of vaccines without any signs of induction of disease activity cannot ultimately be proven by our study design. Lastly, no patients with severe lymphopenia were included, and we can make no statement on the vaccine response in such patients.

In spite of the small number of participants and the limitations mentioned above, we conclude that the seasonal influenza immunization is effective and safe among MS patients treated with DMF.

## Data Availability Statement

The raw data supporting the conclusions of this article will be made available by the authors, without undue reservation.

## Ethics Statement

The studies involving human participants were reviewed and approved by Ethikkommission für das Bundesland Salzburg. The patients/participants provided their written informed consent to participate in this study.

## Author Contributions

TM and PW: design and concept of study, data collection and analysis, and drafting and revision of manuscript. MS, JF, LB, RR, CO’S, GP, AH, KS, EH-B, LM, JG, MR-F, AB, MK, EK, and ET: data collection, and drafting and revision of manuscript. All authors contributed to the article and approved the submitted version.

## Funding

This study was financially supported by Biogen. The funder was not involved in the study design, collection, analysis, interpretation of data, the writing of this article or the decision to submit it for publication.

## Conflict of Interest

Author EK was employed by company Hermesoft. TM received travel support and honoraria for presentations or participation on advisory boards from Biogen Idec, Celgene, Novartis, Roche, Sanofi, Merck, and Teva. ET has received consultation fees and/or speakers honoraria from Arvelle, Argenix, Angellini, Bial, Biogen Idec, Boehringer Ingelheim, Eisai, Epilog, GL Pharma, GW Pharmaceuticals, Ever Pharma, Hikma, LivaNova, Marinus, Medtronics, Newbridge, Novartis, Sanofi, Genzyme, and UCB Pharma. MK has received speaker honoraria from Bayer, Novartis, Merck, Biogen Idec, and Teva Pharmaceutical Industries Ltd. and serves on scientific advisory boards for Biogen Idec, Merck Serono, Roche, Novartis, Bristol-Myers Squibb, and Gilead. He received research grants from Teva Pharmaceutical Industries Ltd., Biogen, and Novartis. AB was trained within the frame of the PhD Program Molecular Medicine of the Medical University of Graz. JF received travel support and honoraria for presentations from Biogen, Merck, Roche, and Sanofi. MS received travel support from Biogen, Merck, Bristol-Myers Squibb, Sanofi, Roche, Teva, and Novartis. PW has received consultation fees and/or speakers honoraria from Bayer, Biogen Idec, Bristol-Myers Squibb, Merck, Novartis, Roche, Sanofi Genzyme, and Teva Pharmaceutical Industries Ltd. He received research grants from Biogen Idec and Merck. EK has received consultation fees from Astra Zeneca, Biogen, Bristol-Myers Squibb, Chiesi, Genzyme-Sanofi, Gilead, Glock, Merck, Novartis Pharma, Pfizer, Roche, Servier, and Vertex.

The remaining authors declare that the research was conducted in the absence of any commercial or financial relationships that could be construed as a potential conflict of interest.

## Publisher’s Note

All claims expressed in this article are solely those of the authors and do not necessarily represent those of their affiliated organizations, or those of the publisher, the editors and the reviewers. Any product that may be evaluated in this article, or claim that may be made by its manufacturer, is not guaranteed or endorsed by the publisher.
